# Erie County Medical Society

**Published:** 1884-05

**Authors:** Edward Clark

**Affiliations:** Secretary


### Erie County Medical Society.

A special meeting of the Erie County Medical Society was held at the Buffalo Medical College April 8, 1884.

Meeting was called to order by the President, Dr. J. C. Greene, who stated that according to the call the meeting was held for the purpose of receiving the report of the Board of Censors relating to pending legislation concerning the incorporation of medical colleges.

There was a large attendance of the city members.

After some delay, as the board was not present to report, Dr. Gay moved to adjourn, but before the motion was put, the Censors made their appearance.

Dr. Storck, Chairman of the Board of Censors, reported, in behalf of the board, that the action taken by the board in the present investigation and preparation of the report was in accordance with instructions received from the society at a former meeting. He stated that much pains and money had been spent in investigating the legality of charters of medical colleges, and that the Board of Censors believed it their duty to treat the present college, viz., Niagara University, the same as they had treated the "Mohawk College," so called.

Dr. Storck then called upon Dr. Hopkins, the secretary of the board, to read the report, which was as follows:

*To the Medical Society of the County of Erie:*

Mr. President and Gentlemen—Your Board of Censors would report that at a meeting of the board held February 20, 1884, certain official communications were received from the secretary of the regents of the university of the State of New York, from which we make the following quotations, viz.:

"The charter to Niagara University was issued under a special statute, passed by the legislature at its last session, Chap. 92, Laws of 1883. The charter does not differ materially from an ordinary college charter. There is no mention, either in the application or the charter, of a medical department. * * * The college will have the same authority to establish a medical department as Rochester University, no more nor less. * * *

"I cannot cite any instance of a college or university now conducting a medical department under a charter granted by the regents except Niagara University."

The above are quotations. After a careful examination of the charter of Niagara University and that of Rochester University to which it is likened by the secretary of the regents, and a full consideration of the correspondence above referred to, the Censors unanimously adopted the following resolution:

"*Resolved*, That the Board of Censors of the Medical Society of the County of Erie hereby request of Messrs. Rogers, Locke & Milburn an opinion upon the authority of Niagara University to conduct a medical department and to grant to its graduates the degree of M. D., the said opinion to be for the information of the said Board of Censors, the expense of the same not to exceed the sum of $25.00."

On the 21st of March the Censors met and received the opinion of Messrs. Rogers, Locke & Milburn, which accompanies this report, and which reaches the conclusion that Niagara University is a literary eollege with authority to grant literary degrees only, and without the authority to grant the degree of M. D.

Inasmuch as the belief prevailed with the Censors that the facts set forth in the legal opinion were competent to establish the reasonableness of its conclusions, it was decided to do what could be done to relieve the members of the medical department of Niagara University from the embarrassments of their situation. Accordingly, the following letters were directed to the medical faculty and the trustees of the above college:

BOARD OF CENSORS OF THE MEDICAL SOCIETY OF THE COUNTY OF ERIE.

BUFFALO, March 21, 1884.

DEAR SIR—The Board of Censors direct me to inform you that at a recent meeting of the board a legal opinion was presented upon the question of the competency of the charter of the corporation known as the Niagara University, of which Board of Trustees I am informed you are a member. In this opinion the Censors were advised that the charter of the said corporation did not authorize the same to confer the degree of M. D. An adjourned meeting of the Board of Censors will be held on the 28th inst., at four o'clock P. M., at the office of Dr. Edward Storck, for the further consideration of this question, and the board will be pleased to hear any communications which you, or the trustees of the Niagara University, may wish to make to them at that time.

Your obedient servant,

H. R. HOPKINS, *Secretary*.

To Rt. Rev. S. V. Ryan, D. D., President Trustees Niagara University.

To which the following replies were received:

March 22, 1884.

*Dr. H. R. Hopkins:*

DEAR SIR—Your communication has been received, and the faculty has referred it to the trustees of the university.

Yours respectfully, A. A. HUBBELL.

BUFFALO, March 21, 1884.

*H. R. Hopkins, M. D., Secretary:*

DEAR SIR—In reply to your favor of this date, I beg to say, that I hardly think it in place for me to say anything to the Board of Censors on the subject in question. The Board of Trustees of the Niagara University believe that they have, from their charter and the Board of Regents, power to confer degrees in all the departments which the University is empowered to teach, and they are fortified in their belief by the best legal advice they could obtain.

Yours most respectfully,

S. V. RYAN, *Bishop of Buffalo.*

This attitude of the parties chiefly interested in the question of the validity of the charter of Niagara University, made up, as it was, of apparent indifference on the one hand and entire confidence on the other, was one not easy to understand.

Shortly after this correspondence between the authorities of Niagara University and the Censors, facts came to light which may have a bearing upon the situation. Under date of March 11th, Mr. Welch, member from Niagara county, introduced a bill to the Assembly, amending the act incorporating the Seminary of Our Lady of Angels. This bill passed that body by unanimous consent upon the 26th of March, two days before the adjourned meeting of the Censors at which the communication of the trustees of Niagara University was received.

This bill now before the Senate is plainly intended to legalize the medical department of Niagara University, and the Censors are advised that in case it should become a law, would have such effect.

At the same time that the attention of the Censors was directed to this bill of Niagara University, their notice was also called to a bill before both houses of the legislature to legalize the past acts of the College of Physicians and Surgeons of Buffalo, and to provide that this college shall have the right to continue as a medical college and to grant the degree of M. D.

The Censors would call the attention of the society to a prominent feature common to both of these bills, which, in our judgment, renders them liable to adverse criticism.

### THE BUFFALO COLLEGE OF PHYSICIANS AND SURGEONS.

SECTION 1. The institution known as the College of Physicians and Surgeons of Buffalo may confer the degree of Doctor of Medicine upon and issue diplomas to such students thereof as shall have pursued in said institution the course of study prescribed by its rules and regulations, and who, after examination by the faculty and trustees of said institution, are entitled to such degree and diploma.

SEC. 2. All medical degrees and diplomas heretofore granted by said institution to the students or graduates of said institution, who shall have pursued in said institution the course of study prescribed by its rules and regulations, and which degrees and diplomas have been conferred after examination entitling said students and graduates to the same, and all degrees and diplomas of such institution which may be conferred and issued under the provisions of this act are hereby legalized and declared to be in all respects valid and of equal sufficiency, force, and effect as medical diplomas, certificates, or licenses granted by any lawfully constituted or incorporated medical society, college, university, or chartered medical school in this State.

SEC. 3. This act shall take effect immediately.

STATE OF NEW YORK.

No. 429.

IN ASSEMBLY.

March 11, 1884—Passed 26th.

Introduced by Mr. Welch—read twice and referred to the committee on public education—reported favorably from said committee, and by unanimous consent ordered to a third reading.

AN ACT

To amend chapter one hundred and ninety of the laws of eighteen hundred and sixty-three, entitled "An act to incorporate the Seminary of Our Lady of Angels," and the acts amendatory thereof.

*The People of the State of New York, represented in Senate and Assembly, do enact as follows:*

SECTION 1. Section six of chapter one hundred and ninety of the laws of eighteen hundred and sixty-three, entitled "An act to incorporate the Seminary of Our Lady of Angels," and as amended by chapter ninety-two of the laws of eighteen hundred and eighty-three, is hereby amended so as to read as follows:

§ 6. Whenever, in the opinion of the regents of the university, the state of literature in the said seminary and the value of its property according to the regulations of the said regents shall justify the same, the said regents may, on the petition of the trustees, by an instrument under their common seal, erect the said seminary into a college with such name, and such number of trustees, and on such conditions, and with such powers and privileges conformable to law as the said regents may deem proper; and the said college shall have the right to maintain any department of learning that is maintained by any college or university in this State, and may locate and maintain any department thereof in the county of Erie; and the trustees of said college shall have the power and right to grant and confer any degree which may be or is granted and conferred by any college, university or other institution of learning in this State legally authorized so to do; and every diploma given in testimony thereof shall entitle the possessor to all the immunities and privileges which by law or usage are allowed to possessors of similar diplomas granted by any college, university or other institution of learning in this State.

§ 2. This act shall take effect immediately.

It will be observed that these bills would confer upon the trustees of their respective colleges the authority to grant the degree of M. D , and that this grant of authority is absolutely without restrictions or conditions of any kind whatever.

The Censors are positively informed that there is not a medical school in the State which has such unconditional powers, but in every case in which a charter has been granted, either to a medical school or to a university with authority to maintain a medical department, certain explicit restrictions and conditions have been placed upon the power to grant the degree of M. D. We are also unable to find any reasons why the trustees of the College of Physicians and Surgeons of Buffalo, or the trustees of the Seminary of Our Lady of Angels, should receive such a grant of authority as has never yet been given to the trustees of any medical school in this State, a grant which gives these trustees absolute, unrestricted power to license physicians to practice the ar- of medicine.

In the legal proceedings instituted by this society against one of these medcal colleges, familiarly known as the Mohawk College, there was one principle kept prominently to the front, a principle which the courts did not fail to recognize and sustain, to wit:

That the responsibilities of a medical college, a body which licenses physicians, are responsibilities of the highest order.

That the legal restrictions imposed by the people upon the formation of such schools are in no manner onerous or difficult of fulfillment.

That the current of professional and of public opinion runs in the direction of raising rather than lowering of such restrictions, and that under no circumstances should such restrictions be disregarded.

In the belief that the facts contained in this report are of such character and moment as merit the special consideration of this society, and for the purpose of receiving such instructions as it may please you to give, the foregoing is respectfully submitted in compliance with a resolution of the Board of Censors.

EDWARD STORCK,
P. W. VAN PEYMA,
H. R. HOPKINS.

After the report was read, Dr. Howe moved its acceptance.

Dr. W. W. Potter moved as an amendment that that the report be received.

Dr. Howe accepted the amendment of Dr. Potter.

Motion, as amended, was adopted.

Dr. Potter asked by what authority the Board of Censors made the present investigation, and asked the secretary to read from the minutes the resolutions giving them such authority.

Dr. Wyckoff suggested that some one who was familiar with the minutes of former meetings would perhaps find the resolutions more quickly than the secretary. Dr. Storck looked over the minutes of previous meetings, but failed to find what he was looking for, and explained to the society that although he could not find the proper authority for the board's action in this matter just at present, that it was given to them by resolutions adopted at a former meeting.

Dr. W. W. Potter moved that as the records failed to show any authority for the present investigation, that so much of the report of the Board of Censors as related to the Niagara University be laid on the table, and called for the ayes and noes. The motion was lost, the vote being as follows:

Ayes—Cronyn, Gay, F. H. Potter, Heath, Daniels, Frederick, Lothrop, Hoyer, W. W. Potter, C. A. Ring, J. L. C. Cronyn, Ingraham, Lynde, Briggs, W. D. Green, Banta, Armstrong, Walsh, Berkes, Jackson, Pattison, Bourne, Dambach, Clark, Davidson, Fell, Willoughby, Kiefer, Hartwig, Runner, Daggett, Tremaine, Stockton, Hubbell, York—35.

Noes—Abbott, King, Putnam, Barrett, Barker, Fowler, Johnson, Wyckoff, Coakley, Brown, E. H. Long, Wm. Ring, Diehl, Mann, Peterson, Mynter, Gregory, Rochester, Howe, McBeth, Hopkins, E. Storck, Cary, Bartow, Folwell, Mackay, Warren, Hauenstein, E. E. Storck, Hebenstreit, Frank, Van Peyma, Keene, Phelps, Niemand, Schade, Wetzel, Hayd, Haberstro, B. G. Long—40.

Dr. Barker asked who were legal voters, and Dr. Storck moved that a recess of five minutes be taken to enable members to pay their dues and become legal voters. Carried.

Meeting was then called to order, and the ayes and noes called for on Dr. Potter's motion which was lost (by the above vote).

Dr. Hopkins stated that two persons, viz.: Dr. Tremaine, (U. S. A.,) and Dr. Heath, (U. S. H. M. S.,) whose names had been called, had not complied with the regulations of the by-laws as applied to members, and consequently had no right to vote.

Drs. Tremaine and Heath said that they supposed themselves to be members; if it was not so they had no desire to vote.

Dr. Hopkins read from section 5 of the by-laws to show that Drs. Tremaine and Heath were not members.

The Chair ruled that Dr. Hopkins' point was well taken.

Dr. Potter moved that inasmuch as the two gentlemen named were already *de facto* members, the rules be suspended and Drs. Tremaine and Heath be now elected to full membership. Carried.

Drs. Barnes and Wall were, by vote, excused from voting on questions before the society.

Dr. Barker offered the following resolutions, and moved their adoption:

WHEREAS, Certain efforts have been made in the legislature of this State which have for their aim the enactment of laws permitting the trustees of certain colleges or universities to grant the diploma of Doctor of Medicine without such restrictions as to age, moral character or professional qualifications as are binding now on all other such institutions in this State; and,

WHEREAS, The statutes already provide for the establishment of such institutions when they conform to certain specified requirements; therefore,

*Resolved*, That the permission to grant such diplomas without the restrictions ordinarily enjoined tends to lower the standard of medical education, and that as such we deem it unwise and think it should be condemned.

*Resolved*, That the Board of Censors be hereby empowered to communicate with the Legislature of this State, and also to transmit to that body a copy of these resolutions.

Dr. Ring offered as an amendment to Dr. Barker's resolution that it apply to the Mohawk College only, for he did not wish the members of Niagara University classed with the men who were endeavoring to run the other institution named. Lost.

Dr. Rochester thought that the resolution should be carefully considered; that it did not apply to any particular institution, whatever its name; that if a college was necessary let it be established on a proper basis, the same as all other medical colleges. If Niagara University is properly established let it live, but I am opposed to granting any college powers which other schools do not possess.

Dr. Cronyn thought that Dr. Rochester did not state the matter fairly; nor did the resolution. Niagara University did not ask for more power than is granted any other medical college. He thought Dr. Hopkins inconsistent when he opposed the Niagara University, for it was an institution of a higher order than many medical schools in the State. A preliminary examination and an extended course of study is required.

Dr. Davidson called attention to the fact that the faculty of the medical department of Niagara University was composed of some of the most honored members of this society, two of them its ex-Presidents. The college was established in Buffalo by an honorable and wealthy corporation, capable of more than complying with the law as to the foundation of universities, in possessing property to the amount of a quarter of a million dollars. The college had under its control the largest hospital in the city and enjoyed superior advantages for clinical instruction. It had bravely raised the standard of its requirements for graduation to those adopted by Harvard and the University of Pennsylvania. Notwithstanding all this, he would ask the society to remember the torrent of invectives and abuse which the new college received from certain members of the society. He had heard it said that the old college did not oppose the Niagara. It was, however, a curious fact that the lying (?) newspapers severally attributed these abusive or unfavorable opinions *only* to certain members of the faculty of the Buffalo Medical College. He also reminded the society how perseveringly certain members of the profession had circulated the story that the charter of the new college was defective. The faculty had acted in good faith, and had, as soon as the charge was made, submitted the question to a legal firm of the highest standing, who had given an unequivocal opinion as to the validity of the charter. To put an end to these injurious and false statements in the quickest way was the object of the trustees in applying for a special act more clearly defining the powers and privileges of the university. He protested against the action of the Censors, in presenting the matter as they had, in that while asking the society to take action against special legislation, especially in reference to the Mohawk College, the society was being dragooned by interested parties into taking part in a conspiracy against the welfare of the Niagara University.

Dr. Tremaine read from the *Express*, of April 5th, extracts from an article embracing the opinion of Rogers, Locke & Milburn as to the legality of the charter of the medical department of Niagara University, and inquired by what authority the Board of Censors had forestalled the action of the society in giving publicity to matter which had not yet been brought before the society, and which was the exclusive property of the society. He considered the publication of the matter a direct insult to the faculty of the Niagara University, and charged the Board of Censors with violation of their duty. He believed the true motive which instigated the publication of the article in question was a desire to injure the Niagara University in the eyes of the people.

In reply to Dr. Tremaine, Dr. Storck disclaimed that the Board of Censors had anything to do with publishing the article embracing the legal opinion of Rogers, Locke & Milburn, which appeared in the *Express* of April 5th. He was sorry that certain statements appeared in the paper, and believed that the reporters were responsible for them.

Dr. Van Peyma believed no distinction should be made between the Niagara University and the Mohawk College; for, said he, "How do we know that the trustees of one school are any more trustworthy than those of the other?"

Remarks were made by several members, and Dr. Stockton moved, as an amendment, that the resolutions offered by Dr. Barker be laid on the table, and called for the ayes and noes. Lost—ayes, 31; noes, 41.

Dr. Barker's resolutions were then adopted, and the meeting adjourned.

EDWARD CLARK, *Secretary.*

