# A Correction

**Published:** 1884-06

**Authors:** E. Storck

**Affiliations:** Buffalo


### Correspondence.

*Editors Buffalo Medical and Surgical Journal:*

GENTLEMEN—In the May number of the JOURNAL, in the report of the proceedings of the meeting of the Erie County Medical Society, a statement appears that Dr. Storck disclaimed that the Board of Censors had anything to do with publishing the article *embracing* the legal opinion of Rogers, Locke & Milburn, which appeared in the *Express* of April 5th. This report is not correct, it should read *prefacing* the legal report. The Board of Censors, all the members present except Dr. Briggs, agreed to have the legal opinion of said law firm published, as the bill in question was then pending before the State Senate. The Board, however, furnished no comments.

An editorial also on the same subject, signed T. L., congratulates the medical profession in this county that two of the five members of the Board of Censors declined to sign the report; this is also incorrect. All the members of the Board were unanimous in the action taken, and agreed to the report as read before the society, except Dr. Briggs, who never met with the Board.

Have the kindness to make these corrections, and oblige,

Yours respectfully,

E. STORCK.

BUFFALO, May 21, 1884.

